# The complete mitochondrial genome of *Cynoglossus joyneri* (Teleostei: Pleuronectiformes)

**DOI:** 10.1080/23802359.2016.1192512

**Published:** 2016-11-12

**Authors:** Li-Hua Ren, Tao Xu, Guo-Hua Sun, Fan Li, Wei-Jun Wang

**Affiliations:** a Shandong Marine Resource and Environment Research Institute, Yantai, China;; b Shandong Fishery Technology Extension Center, Jinan, China

**Keywords:** Fish, three-lined tongue sole, Cynoglossidae, paraphyly

## Abstract

The complete mitochondrial genome of a commercially important sea fish *Cynoglossus joyneri* was sequenced and annotated. The 16,941 bp-long genome contained 13 protein-coding genes, 2 ribosomal RNAs, 22 transfer RNAs, and a control region. Phylogenetic analysis indicated that *C. joyneri* is a sister group with *C. sinicus* and *C. bilineatus*, and corroborated the proposed paraphyly of *Cynoglossus* genus.

Three-lined tongue sole *Cynoglossus joyneri* (Cynoglossidae) is a commercially important (Baeck et al. 2011), but relatively poorly researched demersal saltwater fish native of north-western Pacific. Cynoglossidae are interesting both from evolutionary and taxonomic perspective, as the family is fast-evolving (Pardo et al. 2005) and paraphyletic (Xu et al. 2008). Publication of the complete mitochondrial genome of this species shall be useful for future researches about the evolution and taxonomy of this family, as well as for the fisheries management.

Fin tissue sample was collected from a specimen (stored in the Shandong Marine Resource and Environment Research Institute, accession number 20150801CJ02) captured in the Laizhou bay, Bohai Sea, China (119°08’ E, 37°16’ N). Twenty two primer pairs were used to amplify the entire mitochondrial genome sequence (GenBank accession number: KU497492). The total size (16,941 bp) is comparatively large among fishes (Guo et al. 2004), but the organization is standard: 13 protein-coding genes, 22 tRNA genes, two rRNA genes (*12S* and *16S*), and one control region (D-loop).

Eight tRNAs and *NAD6* gene were encoded on the H-strand. Most protein-coding genes had the standard start (AUG) and stop (UAG and UAA) codons. Exceptions were *COX1* gene – initiated by the GUG codon, *NAD4* – terminated by AGA, and *COX2* and *NAD3* – terminated by U – (completed by the addition of A residues to the mRNA). The mitogenome exhibited a relatively strong (57.4%) A + T bias (A = 29%, T = 29%, G = 15%, C = 27%).

Both approaches used to estimate the phylogenetic position of *C. joyneri* produced identical dendrogram topologies: maximum-likelihood estimation (RaxmlGUI, GTR + G + I, 1000 bootstrap replications; Silvestro & Michalak 2012) and Bayesian inference (MrBayes 3.26, default settings, four MCMC chains, GTR + G + I, 5 × 10^6^ generations; Ronquist & Huelsenbeck 2003). All available Cynoglossidae mitochondrial genome sequences (14), and *Zebrias crossolepis* (Pleuronectiformes: Soleidae) as outgroup, were retrieved from GenBank. Analyses were performed on concatenated 13 mitochondrial protein-coding genes and two rRNAs (adding up to 14,246 bp). Dendrogram topology shows that *C. joyneri* forms a sister group with *C. sinicus* and *C. bilineatus*, it corroborates the proposed paraphyly of *Cynoglossus* genus (Xu et al. 2008), and indicates that *C. punticeps* and the entire *Paraplagusia* genus have relatively recently split from the common ancestor (Figure 1).

**Figure 1.**
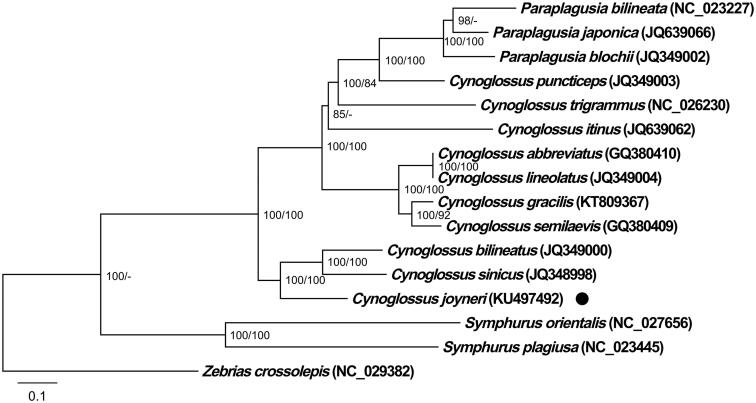
Phylogenetic dendrogram showing the evolutionary relationships among Cynoglossus joyneri (highlighted by a black dot), 14 Cynoglossidae species, and Zebrias crossolepis (Soleidae, Pleuronectiformes) as outgroup. GenBank accession numbers are indicated in the figure. Maximum-likelihood and Bayesian analyses were performed using partial genomes. Scale bar corresponds to the estimated number of substitutions per site. Bootstrap support (left) and Bayesian posterior probability (right) values are displayed next to the nodes. Values below 70 are displayed as -.
